# A Quest for miRNA Bio-Marker: A Track Back Approach from Gingivo Buccal Cancer to Two Different Types of Precancers

**DOI:** 10.1371/journal.pone.0104839

**Published:** 2014-08-15

**Authors:** Navonil De Sarkar, Roshni Roy, Jit Kumar Mitra, Sandip Ghose, Arnab Chakraborty, Ranjan Rashmi Paul, Indranil Mukhopadhyay, Bidyut Roy

**Affiliations:** 1 Human Genetics Unit, Indian Statistical Institute, Kolkata, India; 2 Oral Pathology Department, Guru Nanak Institute of Dental Science & Research, Panihati, Kokata, India; Saint Louis University, United States of America

## Abstract

Deregulation of miRNA expression may contribute to tumorigenesis and other patho-physiology associated with cancer. Using TLDA, expression of 762 miRNAs was checked in 18 pairs of gingivo buccal cancer-adjacent control tissues. Expression of significantly deregulated miRNAs was further validated in cancer and examined in two types of precancer (leukoplakia and lichen planus) tissues by primer-specific TaqMan assays. Biological implications of these miRNAs were assessed bioinformatically. Expression of hsa-miR-1293, hsa-miR-31, hsa-miR-31* and *hsa-miR-7* were significantly up-regulated and those of *hsa-miR-206, hsa-miR-204* and *hsa-miR-133a* were significantly down-regulated in all cancer samples. Expression of only *hsa-miR*-31 was significantly up-regulated in leukoplakia but none in lichen planus samples. Analysis of expression heterogeneity divided 18 cancer samples into clusters of 13 and 5 samples and revealed that expression of 30 miRNAs (including the above-mentioned 7 miRNAs), was significantly deregulated in the cluster of 13 samples. From database mining and pathway analysis it was observed that these miRNAs can significantly target many of the genes present in different cancer related pathways such as “proteoglycans in cancer”, *PI3K-AKT* etc. which play important roles in expression of different molecular features of cancer. Expression of *hsa-miR-31* was significantly up-regulated in both cancer and leukoplakia tissues and, thus, may be one of the molecular markers of leukoplakia which may progress to gingivo-buccal cancer.

## Introduction

Gingivo buccal squamous cell carcinoma (GBSCC) is one of the most prevalent (∼60%) cancers in oral cavity, especially among the tobacco users in India. Entire set of head and neck squamous cell carcinoma stands as the fifth most common malignancy worldwide [1]. But head and neck cancer comprises ∼24% of total cancers in India as recorded at a tertiary hospital, Tata Memorial Centre, Mumbai and about ∼13.5% of them are from the oral cavity [2]. Five years survival rate (∼50%) of patients suffering from head and neck squamous cell carcinoma has not improved much even after intense research during last 15 years, so, early detection is still a key issue for better survival [3].

Since 1993, miRNA has emerged to be one of the most prominent biological regulators, which play important roles in controlling and fine tuning its target's (mRNA) expression [4]–[9]. In recent years, it has been demonstrated that microRNAs (miRNAs) are also involved in human tumorigenesis and could act either as tumorigenic/oncogenic or anti-tumorigenic molecules. Thus, it is making a new layer in the molecular events in human cancer. Gene expression studies revealed that many miRNAs are deregulated in different cancer types and functional studies clarified that miRNAs are involved in several molecular and biological processes that drive tumorigenesis [10]–[12]. Thus, in addition to different scales of variability in terms of new mutations in genome or genetic background of the patients (i.e. germ line mutation), variability in expression of different miRNAs is also a major aspect for investigation. With this belief, we studied expression deregulation of 762 miRNAs in GBSCC and also checked whether similar kind of expression deregulation could be observed in precancerous leukoplakia and lichen planus tissues from oral cavity. Efforts have been made to understand how these miRNAs may be involved in cancer using pathway analysis and information from lit*e*ratures and databases.

## Materials and Methods

### Samples

This study was approved by “Review committee for protection of research risk to humans, Indian Statistical Institute”. All individuals in this study have provided written informed consents to publish case details. All participants signed a questionnaire containing demography, tobacco habit and a statement describing that he/she has no objection for use of blood and tissue samples in this study and is participating in this study voluntarily. Unrelated patients suffering from cancer (n = 18), lichen planus (n = 12) and leukoplakia (n = 18) were considered for this study from Guru Nanak Institute of Dental Science and Research, Panihati, Kolkata, (Table 1, Table 2, Table 3). One tissue punch from cancer/precancer site and another punch from adjacent “clinically” normal site (at least 1.5 inch away from the border of the lesion) were biopsied by oral pathologist. One portion of biopsy tissues was used for histopathological confirmation and remaining part of tissues were kept separately in “RNA Later” at −80°C and used within 2 months.

**Table 1:** Demography of 18 cancer patients and their tumor differentiation status.

*Sample	Site	age	sex	histopath observation	Clinical TNM
S1	**Right Buccal Mucosa(Cheek)**	39	M	Well diff	T2N0Mx@
S2	**Cheek**	55	M	Well diff	T2N1Mx
S5	**Right Cheek**	65	F	Mod. diff (transition)	T2N1Mx
S6	**Right Lower Buccal Mucosa**	60	F	Well diff	T2N1Mx
S7	**Cheek**	40	M	Well diff	T1NxMx
S8	**Cheek**	45	M	Well diff	T1N0Mx
S11	**Gingiva**	52	M	Mod. diff (transition)	T1N0Mx
S12	**Right Gingiva**	45	F	Well diff	T1NxMx
S13	**Left Buccal Mucosa(Cheek)**	60	F	Well diff	T2N1Mx
S15	**Right Buccal Mucosa(Cheek)**	45	F	Well diff	T2N1Mx
S16	**Left Cheek**	40	F	Well diff	T2NxMx
S17	**Right Retro-molar Region**	51	M	Well diff	T1N1Mx
S19	**Left Buccal vestibule**	68	F	Well diff	T1N1Mx
S20	**Left Buccal Mucosa(Cheek)**	66	M	Well diff	T1N1Mx
S21	**Left Buccal Mucosa(Cheek)**	45	M	Well diff	T2N1Mx
S26	**Gingiva**	40	M	Well diff	T2N1Mx
S27	**Cheek**	55	F	Well diff	T2N0Mx
S24	**Cheek**	80	M	Well diff	T3N2cMx

*All patients had tobacco habits;

@ Mx: metastasis status not known.

**Table 2:** Demography of 12 lichen planus patients.

*Sample	Site	age	sex	Clinical Type	Histopath Report
LP1	Both Buccal Mucosa(Cheek)	55	M	plaque	Oral lichen planus
LP2	Right Buccal Mucosa	26	M	Reticular	Oral lichen planus
LP3	Right Buccal Mucosa	45	F	Erosive	Oral lichen planus
LP4	Right Buccal Mucosa	52	F	Reticular	Oral lichen planus
LP5	Both Buccal Mucosa	25	F	Reticular	Oral lichen planus
LP6	Both Buccal Mucosa and Gums	45	F	Erosive	Oral lichen planus
LP7	Right Buccal Mucosa	37	F	Reticular	Oral lichen planus
LP8	Both Buccal Mucosa	35	F	Reticular	Oral lichen planus
LP9	Both Buccal Mucosa	45	M	Reticular	Oral lichen planus
LP10	Both Buccal Mucosa	48	M	Erosive	Oral lichen planus
LP11	Both Buccal Mucosa	36	F	Reticular	Oral lichen planus with mild epithelial dysplasia
LP12	Both Buccal Mucosa	50	F	Reticular	Oral lichen planus

*2 out of 12 (Patient LP1 and LP6) patients had tobacco habits.

**Table 3:** Demography of 18 leukoplakia patients.

*Samples SSampleSample	Site	age	sex	Histopath Report
LK1	**Left Buccal Mucosa(Cheek)**	53	M	Mild epithelial dysplasia
LK2	**Buccal Mucosa**	55	M	Mild epithelial dysplasia
LK3	**Buccal Mucosa**	56	M	Mild epithelial dysplasia
LK4	**Both Buccal Mucosa**	32	M	Mild epithelial dysplasia
LK5	**Right Buccal Mucosa**	49	M	Mild epithelial dysplasia
LK6	**Both Buccal Mucosa**	45	M	Mild epithelial dysplasia
LK7	**Both Buccal Mucosa**	32	M	Moderate epithelial dysplasia
LK8	**Left Buccal Mucosa**	42	M	Mild epithelial dysplasia
LK9	**Both Buccal Mucosa**	44	M	Mild epithelial dysplasia
LK10	**Both Buccal Mucosa**	30	M	Mild epithelial dysplasia
LK11	**Right Buccal Mucosa**	46	M	Moderate epithelial dysplasia
LK12	**Both Buccal Mucosa**	61	M	Mild epithelial dysplasia
LK13	**Both Commissural Area**	48	M	Mild epithelial dysplasia
LK14	**Both Buccal Mucosa**	35	M	Mild epithelial dysplasia
LK15	**Both Buccal Mucosa**	25	F	Mild epithelial dysplasia
LK16	**Both Buccal Mucosa**	40	M	Mild epithelial dysplasia
LK17	**Right Buccal Mucosa**	54	M	Mild epithelial dysplasia
LK18	**Both Buccal Mucosa**	34	M	Mild epithelial dysplasia

*All except two (LK5 & LK9) patients had tobacco habits.

### RNA isolation and TLDA assay

RNA isolation was done using mirVana kit (Life Technologies, USA). RNA yield quantification was done by Nano Drop and integrity was checked with Agilient's Bioanalyzer (Agilent RNA6000 Nano Kit), respectively. RIN number cut-off was chosen as ≥6.9. Parallel expression assay of 762 miRNAs was performed using TLDA-A (V2) and TLDA-B (V3) card in 7900HT FAST Real Time PCR system (Applied Biosystems, USA) using TLDA flat block [13]. Primary analysis was done using SDS and Data Assist (Life Technologies, USA) software packages to get expression in terms of Ct, ΔCt and ΔΔCt values where Ct =  Cycles at which the PCR product quantity reaches a defined threshold, ΔCt =  Ct _of a miRNA in cancer tissue_ - Ct _of geometric mean of expression of 3 most stable endogenous control miRNAs in that tissue_ and ΔΔCt =  ΔCt _of a miRNA in cancer tissue_ - ΔCt _of that miRNA in control tissue_. Out of the assayed 762 miRNAs, five were candidates for endogenous controls. However on the basis of their expression stability across all samples, RNU-48, RNU-44 and U6/mmu-6 were selected as endogenous controls. To get ΔCt as the normalized measure of expression of a miRNA in both cancer/precancer and control tissues, expression of all miRNAs in tissues was normalized independently using geometric mean of expression of selected 3 endogenous control miRNAs in the same tissue. Ct value less than 40 were only considered for further analysis.

### Analysis

#### Data Pruning

If expression of any particular miRNA was observed to be present in at-least 9 out of 18 cancer-normal paired tissues, then only, it has been considered for further downstream analysis. Annotation of TLDA assay V2 (A-card) and V3 (B-card) follows miRBase release-14 [13]–[14]. Now, expression data of those miRNAs were considered for further analysis whose annotation is still valid in miRBase release-19 [15]–[16]. In this way expression of 531 miRNAs was considered for subsequent down-stream analysis.

#### Statistical Analysis

Initially, one sample Kolmogorov-Smirnov (KS) test for expression data of all 531 genes was performed independently so as to check for normality of the differential expression values across all samples [(Zi =  {(Ci-Ni)-(mean C- mean N)}, so Zp =  ∑Zi/SD Z, KS test was done on Zp; Ci: i^th^ Cancer sample's ΔCt value, Ni: i^th^ Normal sample's ΔCt value] and eventually expression was found to be normally distributed. It is to be noted that ΔCt values are already log transformed, so, no further log transformation was done with this set of expression data. Followed by the normality test, one tailed paired t-test was performed [(Ci-Ni)>1/<1]. Null hypothesis of one tailed paired t-test was “expression of a particular miRNA is not greater than 2 fold up/down regulated”. So naturally, alternative hypothesis was “expression of a particular miRNA is >2 fold up/down regulated. Test for up-regulation (lower tail test) was performed if the median of a particular miRNA's ΔΔCt is less than zero. Similarly, test for down-regulation was performed if the median of a particular miRNA's ΔΔCt is more than zero. Multiple testing corrections using Benjamini-Hochberg method was performed at 5% level of significance [17] and corrected cut off p-value was 0.00065.

#### Cluster Analysis of miRNAs expressed in cancer tissues

K-median clustering method was used for the entire pruned data set to see whether genome wide miRNA expression variations between individuals were large enough to reliably divide the samples into a number of sub clusters. For this clustering, chosen distance metric was Euclidean. Since, expression of many miRNAs was absent in our data set, it was creating “Absent data” situation. This situation is known to bias the clustering if the most popular clustering method- K-means was adopted. So, the choice of clustering was K-median. The number of reliable clusters, the data would form, was determined using “the elbow” method.

#### TaqMan Assay

Expression of significantly de-regulated miRNAs, detected in cancer tissues by TLDA method, were also validated in same cancer tissues and examined in leukoplakia and lichen planus tissues by TaqMan assay (7900HT Fast Real Time PCR system, Applied Biosystems, USA). Probes and primers were supplied by Invitrogen India Ltd and data were retrieved as “fold change” compared to adjacent controls. Normalization of expression of each gene in each sample was done using geometric mean of the same 3 endogenous controls, viz. *RNU-44, RNU-48* and *mmu-6*, to get ΔΔCt value of that miRNA.

## Results

### Expression profile in cancer tissues by TLDA

**Figure 1:**
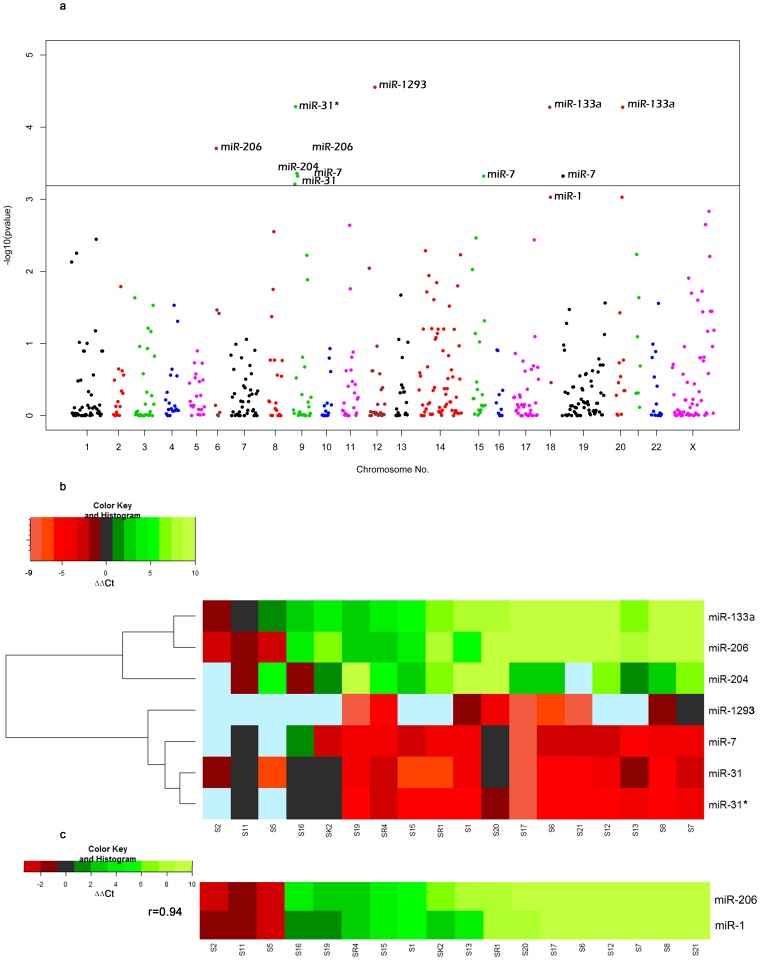
**A: Manhattan plot of p-values for all 528 miRNAs 18 samples.** The AB-line (i.e. horizontal line in the middle of Figure) represents P-value cut off, p = 0.00065. Relative location of 528 miRNAs (along the horizontal axis) across the human genome and their corresponding −log_10_ transformed p-value (along the vertical axis) was plotted. **B: Heat map diagram of ΔΔCt values.** Two-way unsupervised hierarchical clustering of 7 miRNAs whose expression was significantly deregulated samples. Each row represents expression of a miRNA and each column represents a sample. Sky-blue colored cells stand for failed assay i.e. no data in those cells. Red and green colors signify up- and down-regulation, respectively. Dendogram along the vertical axis represents hierarchical classification of miRNAs on the basis of expressions similarity. Distance metrics of hierarchical clustering was Euclidean distance. Heat map was constructed using Heatmap 2 of R's “gplot” package. **C: Highly correlated expression of** ***miR-206*** **and** ***miR-1***.

Expression data of 762 miRNAs were assayed by this method but after data pruning, expression data of 531 miRNAs (including 5 endogenous controls) was used for other down-stream analysis (File S1). During statistical test, at least 2-fold expression change in cancer tissue compared to its paired-control tissue was considered to be the bench-mark of expression deregulation. Thus, expression of 7 miRNAs was found to be significantly deregulated after multiple test correction and all of these seven miRNAs had >4-fold average expression deregulation (i.e. either up- or down-regulation). Validation of expression by miRNA specific TaqMan assay also reconfirmed expression deregulation in cancer tissues although fold-changes were diminished compared to TLDA outcome (Table 4). The difference in sensitivity of these two RT-PCR based (TLDA and miR-specific TaqMan Assay) experimental methods, coupled with the fact that these two sets of experiments were performed at two different time points, might be the influencing factors for difference in degrees of expression deregulation of miRNAs. Relative locations of these 7 miRNAs along with other miRNA genes across different chromosomes revealed that *hsa-miR-133a* and *hsa*-*mir-7* are located on two (Chr 18 and Chr 20) and three chromosomes (Chr 9, Chr 15 and Chr 19), respectively (Figure 1a). Here, assays were performed for only mature miRNAs, thus, expression of *hsa-miR-133a* and *hsa*-*mir-7* might be cumulative sum of all mature forms of respective miRNAs. Among these 7 miRNAs, expression of 4 miRNAs viz. hsa-miR-1293, hsa-miR-31, hsa-miR-31* and *hsa-miR-7* were significantly up-regulated and those of 3 miRNAs viz. *hsa-miR-206, hsa-miR-204* and *hsa-miR-133a* were significantly down regulated in cancer samples (Table 4). Heat map was constructed according to two ways unsupervised hierarchical clustering. So, all down-regulated miRNAs clustered together at the upper part of the plot whereas all up-regulated miRNAs clustered at the bottom (Figure 1b). It showed values of expression (i.e. ΔΔCt) compared to adjacent control as well as number of samples providing expression data. Expression of *hsa-mir-1293* was obtained from 9 cancer-control paired samples but 6 of them had ΔΔCt values ≤−2 and remaining 3 samples had ΔΔCt values between 0 and −2. So, for *hsa-mir-1293*, all these 9 samples had ΔΔCt values with -ve sign, meaning; whenever expression was obtained, it is always up-regulated but 6 of them showed more than 4-fold expression change. Similarly, expression data for hsa-mir-31* were obtained from 16 cancer-control paired samples. Out of these 16 samples, one sample had ΔΔCt between 0 & +2, two samples had ΔΔCt values between 0 & −2 and remaining 13 samples had ΔΔCt values ≤−2.

**Table 4:** Expression profile of 7 miRNAs in precancers and cancer samples.

	Cancer (TLDA) (n = 18)	Cancer (Taqman)(n = 18)	Leukoplakia (n = 18)	Lichen planus (n = 12)
Hsa-mir-	Average fold # change	Average fold # change	Average fold change	Average fold change
1293	28.72↑	4.99↑	1.18	1.32
7	8.3↑	3.89↑	1.18	1.25
31	10.69↑	5.37↑	@4.55 ↑	1.42
31*	13.64↑	6.73↑	4.75↑	1.23
204	23.59↓	27.02↓	1.99↓	1.35
206	65.09↓	31.54↓	1.34	1.23
133a	103.7↓	97.14↓	1.46	1.39

# Expressions of all miRNAs are significantly deregulated in cancer samples after multiple corrections.

@Expression of only miR-31 is significantly deregulated in leukoplakia.

↑ : Upregulation;

↓ : Downregulation.

**Figure 2:**
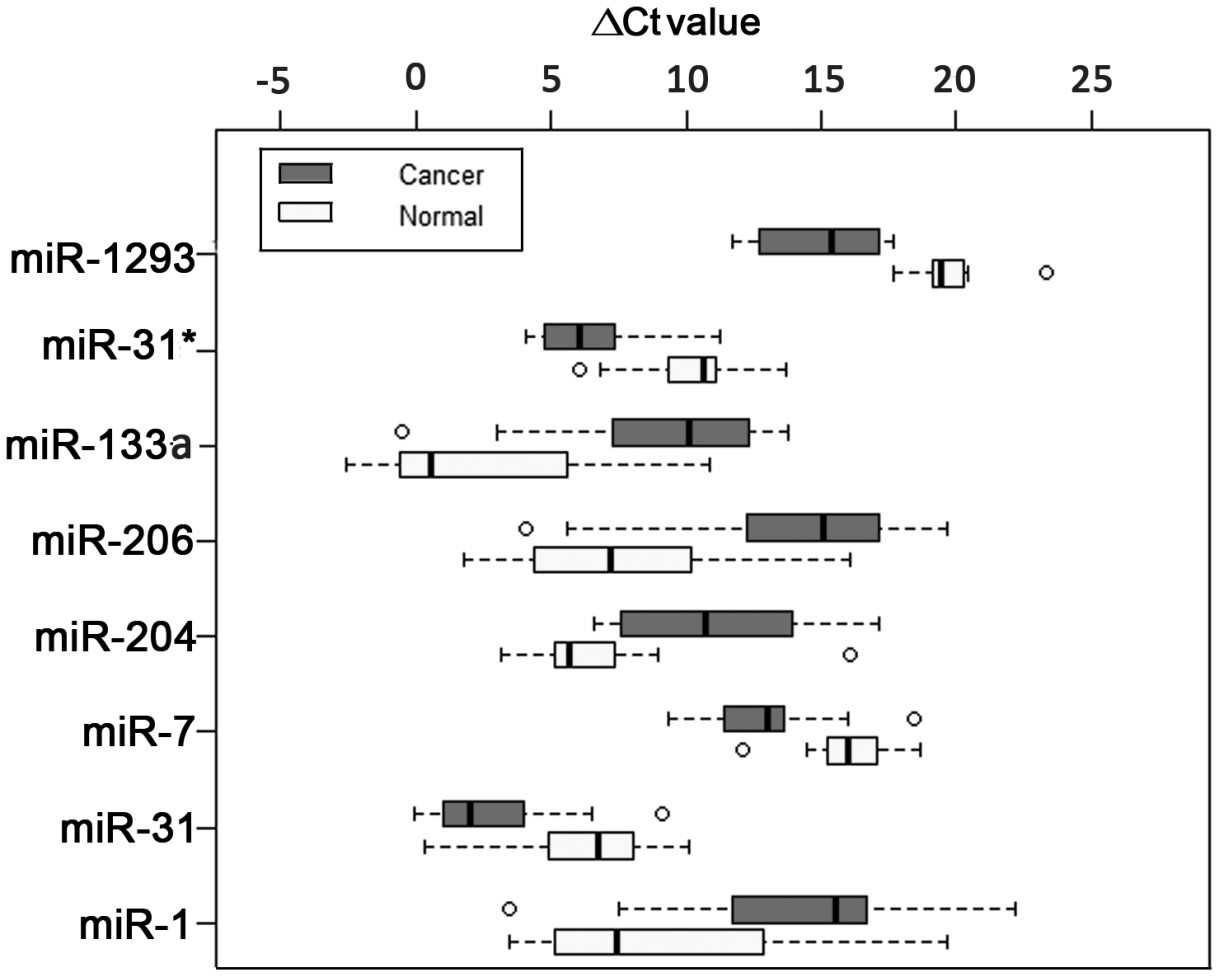
Forest plot showing expression variability of 8 miRNAs. Horizontal box plot is shown for distribution of expression (ΔCt) of miRNAs along the horizontal axis. P-values of miRNAs are plotted in ascending order (top to bottom). Expression of *has-miR-1* is not significantly deregulated and shown to compare with those of other 7 miRNAs. Each Box and whisker pair (Gray for Cancer and white for Normal) represents range of variability of ΔCt values for a miRNA from TLDA data. This range represents expression of all successfully assayed samples data for that miRNA.

Normalized expression (ΔCt) of these 7 miRNAs in cancer and control tissues were mostly non-overlapping and ΔCt values of different miRNAs across the control tissues also showed quite a wide range of variation (Figure 2). So to get correct relative expression, it is important to compare expression of miRNAs in cancer tissue with those of control tissue from the same individual. In this figure, miRNAs were placed according to increasing p-values (from top to bottom) vertically. The ΔCt values of 8 miRNAs in different tumor and control samples had been plotted on the horizontal axis to show distribution of ΔCt values across all the samples. It is evident that more the overlap between ranges of expression of miRNAs in cancer and control tissues, less is the level of significance. Here, *hsa-mir1293* with lowest p value 0.000028 had been positioned on the top and *hsa-mir-1* with p value of 0.00094 (just below the corrected level of significance) was placed at the bottom on the vertical axis (Figure 2).

### Expression of miRNAs in precancerous leukoplakia and lichen planus samples

Among 7 miRNAs which was observed to be significantly deregulated in cancer samples (Table 4), expression of only *miR-31* was significantly up-regulated in leukoplakia tissues where as expression of none of these 7 miRNAs was deregulated significantly in lichen planus tissues. Expression of miR-31* and *miR-204* was also up- (4.75 folds) and down-regulated (1.99 folds), respectively, in leukoplakia tissues but not significantly different.

### Database mining and bioinformatics analyses

Published reports on these 7 miRNAs had also shown similar direction of expression deregulation in some cancers including head and neck [10], [18]–[20]. A total of 561 unique targets were identified when these 7 miRNAs were used to search targets using miRWalk [21] and further cross-validated from Pubmed (http://www.ncbi.nlm.nih.gov/pubmed). The hsa-miR-31* has validated target, *RhoA*, which is reportedly implicated in mouth neoplasm [18]. The *hsa-miR-1293* till now is known to target *GCN1L1* (Table 5) and *hsa-miR-1293* mediated down regulation of this tumor antigen gene (i.e. *GCN1L1*) could contribute to poor prognosis of the tumor [22]. IPA tool was used for “disease term search” (Ingenuity® Systems, www.ingenuity.com) and most significant “disease term” in cancer category of IPA was “head and neck” cancer. IPA could not provide any hit by *hsa-miR-1293* and considered *hsa-miR-31* and hsa-miR-31* as synonymous so the output was from 5 miRNAs. It was also noticed that IPA considered *hsa-miR-206* synonymous to *hsa-miR-1* since they have identical seed sequence [23]. It was also observed that expression deregulation status of *hsa-miR-1* and *hsa-miR-206* across the samples were very similar to each other (r = 0.94) (Figure 1c). Inclusion of *hsa-miR-1* in target search list increased the number of targets to 702. In KEGG pathway mapping portal [24]–[25] validated targets of these 8 miRNAs have been used. Top most pathways in the mapped list were “microRNAs in cancer” followed by “proteoglycans in cancer” and “global cancer pathway” (Table S1 in File S2). Other top relevant pathway was *PI3K-AKT* which is one of the most common pathways implicated in cancer. Other most significant changes that could have happened due to these miRNAs are disruption of actin cytoskeleton maintenance and focal adhesion. Other probable implicated signaling pathways were *RAS*, *RAP1, MAPK, HIF-1*, *FOXO, TNF, ErBB*, apoptosis etc (Table S1 in File S2). “GO” term enrichment search was performed using input of 702 validated targets of these eight miRNAs (data not shown) and it was observed that most prominent disrupted biological processes are primarily related to cell migration, loss of apoptosis, cell proliferation etc. But DAVID based “GO” term enrichment for biological process showed most prominent biological process is “Apoptosis” (Table S2 in File S2) [26]–[28]. All these observations support that these 8 miRNAs may play important functional roles in gingivo buccal cancer. Our RNA-Seq data shows that expression of *FN1, MSN* and *MMP9*, which belongs to “Proteoglycans in cancer” gene list and are targets of down-regulated miRNAs, was up-regulated in 10 of 13 tissue samples (on average, 6.83, 2.43 and 21.89 folds, respectively, compared to adjacent normal). Similarly predicted up-regulation of *LAMC* and down regulation of *PAI*, which belong to *PI3K-AKT* pathway, was also validated in RNA-Seq data in a sub set of these tissue samples (unpublished data). These observations also support our predictive pathway analysis regarding involved biological process/pathways that could be targeted by this set of 8 miRNAs.

**Table 5:** Reported targets associated with 7 miRNAs significantly deregulated in 18 cancer samples.

miRNA expression and p-values for test of significance				Report in previous Publications	
miR	∧p-value	Average ΔΔCt	Median ΔΔCt	Deregulation	Validated Report
*hsa-miR-1293*	2.8E^−05^	−4.84 ^@^	−5.17 ^@^	Up	*GCN1L1*
hsa-miR-31*	5.2E^−05^	−3.77 ^@^	−4.74 ^@^	Up	MN
*hsa-miR-133a*	5.3E^−05^	6.70 $	6.31 $	Down	HN,SCC, LN, EN
*hsa-miR-206*	1.97E^−04^	6.02 $	7.07 $	Down	NEO-NOTCH3 inhibitin., LN
*hsa-miR-204*	4.39E^−04^	4.56 $	4.45 $	Down	HN metastasis Suppressor
*hsa-miR-7*	4.78E^−04^	−3.05 ^@^	−3.18 ^@^	Up	MN (RECK)
*hsa-miR-31*	6.18E^−04^	−3.42 ^@^	−3.54 ^@^	Up	MN,LN, SCC,HN,OLP,EN
# *hsa-miR-1*	9.4E^−04^	3.67 $	5.07 $	Down	

@ : Up-regulation of expression of miRNAs.

$ : Down regulation of expression of miRNAs.

∧ Benjamini-Hochberg corrected p-value cut off at 5% level: 6.5E^−04^.

**ΔΔCt** =  ΔCt _of a gene in cancer tissue_ - ΔCt _of that gene in control tissue_.

# ; Expression of *has-miR-1* is not significantly deregulated and shown for comparison only.

MN- Mouth Neoplasm, HN- Head and Neck Cancer, SCC- Squamous Cell Carcinoma, LN- Laryngeal Neoplasm, EN- Esophageal Neoplasm, OLP-Oral Leukoplakia, *GCN1L1*- general control of amino-acid synthesis 1-like 1.

**Figure 3:**
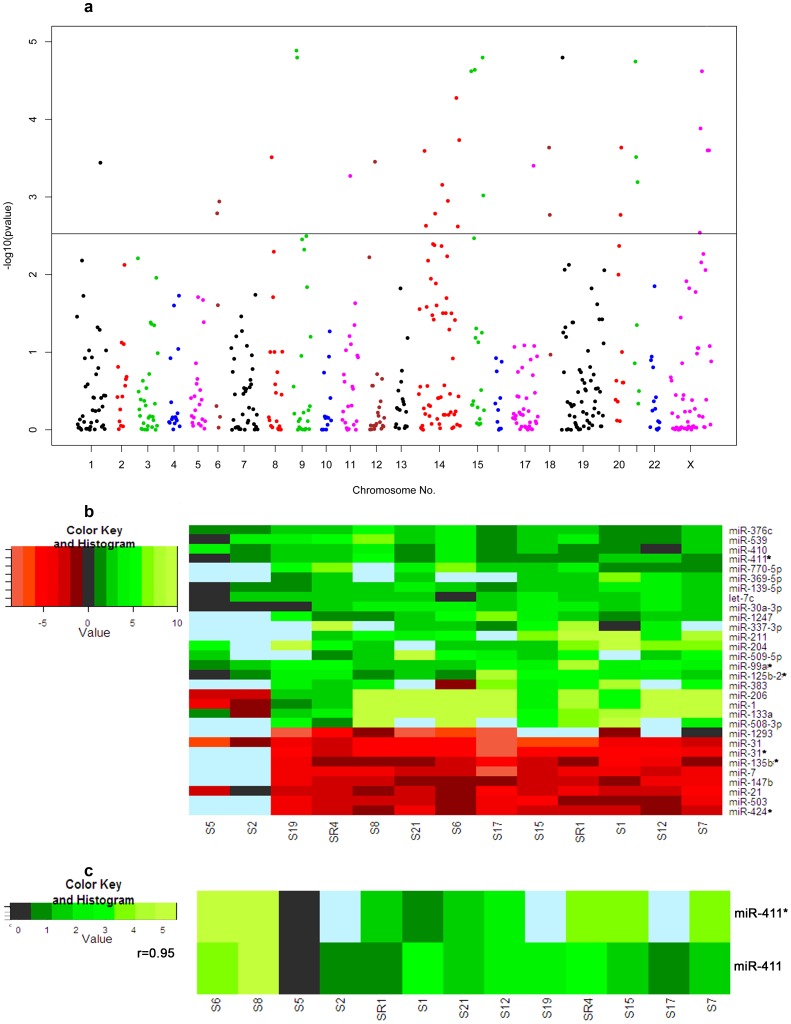
**A: Manhattan plot of p-values for 520 miRNAs from the cluster of 13 samples.** The plot of relative location of 520 miRNAs (along the horizontal axis) across the human chromosome and their corresponding –log_10_ transformed p-value (along the vertical axis). Benjamini-Hochberg corrected P-value cut off was 0.00298 (Horizontal line in the middle of figure). **B: Heat map diagram of ΔΔCt values of 30 miRNAs**. Expression of these miRNAs was significantly deregulated in the cluster of 13 samples. Each row represents a miRNA and each column represents a sample. Sky-blue colored cells stand for failed assay (i.e.no data in those cells). Red and green colors signify up- and down-regulation of expression, respectively. Heat map was constructed using Heatmap 2 of R's “gplot” package. C: Highly correlated expression of miR-411* and miR-411.

Selected samples had little variation in terms of its differentiation status or clinical staging. Cluster analysis was performed to check whether genome wide miRNA profile could explain such characteristic variation. It was performed using expression deregulation data (ΔΔCt) of 531 miRNAs from 18 cancer samples using K-median method. Optimally, only two distinct clusters were obtained; one consisted of 13 paired tumor-normal samples and other consisted of 5 paired tumor-normal samples. It was evident that these two clusters of samples were not formed on the basis of their differentiation status or clinical stages. Expression of 30 miRNAs was significantly deregulated in the cluster of 13 samples (p-value cut off 0.00298 after Benjamini-Hochberg Correction, Figure 3a, Figure 3b) but none of the miRNAs were significantly deregulated in the cluster of 5 samples (data not shown). Fold expression (up- or down-regulated) of a miRNA in a tumor tissue compared to its adjacent control and number of samples providing expression data of a miRNA could be observed in Heat map diagrams of cluster of 13 samples (Figure 3b). It showed that expression of all miRNAs was not available from all samples. Expression of some miRNAs was up-regulated in most of the samples (e.g. expression of miR-31*, miR-7, miR-21 shown at the bottom of the figure) and expression of some miRNAs was down-regulated in most of the samples (e.g. *miR-206, miR-1, miR-133a* shown at the middle of the figure) (Figure 3b).

Out of these 30 miRNAs (Table 6), expression of 28 miRNAs showed similar expression deregulation pattern as it was already reported in earlier studies on cancer [10], [29]–[30]. Of the remaining two miRNAs, one miRNA *(hsa-miR-770-5p*) has not been associated yet with any cancer. Expression of remaining one miRNA (*hsa-miR-211*) has been deregulated in opposite direction in this study compared to previous reports on colorectal cancer [29]–[30]. Actually, 7 miRNAs, whose expression was significantly deregulated in the analysis of 18 samples, are a subset of these 30 miRNAs. However, expression of hsa-miR-411* was observed to be significantly deregulated in this study, but no previous report had shown its involvement with cancer. Again, it is known that *hsa-miR-411* and miR-411* are originated from the same precursor miRNA and *hsa-miR-411* has a strong association with cancer [31]. When we checked expression of mature *hsa-miR-411* and hsa-miR-411*, a strong positive correlation was observed (r = 0.95) (Figure 3c) although expression of *hsa-miR-411* was not found to be statistically significant. Similarly, we have observed significant expression deregulation of hsa-miR-135b* and *hsa*-miR-99a* and reports of association of cancer with *hsa-miR-135b* and *hsa*-*miR-99a* [32]. Searching in similar way, 1207 unique target genes were obtained for these 30 miRNAs. IPA and KEGG mapping were performed using these 30 miRNAs and their known 1207 target genes. In IPA analysis, three cancers, which were found to be associated with expression deregulation of a major subset of these miRNAs, were hypo pharyngeal, esophageal and head and neck cancer (p-values 1.71×10^−07^, 2.20×10^−07^ and 1.02×10^−07^ respectively). Interestingly, same biological signaling pathways (relevant to cancer) were reported to be involved with these set of 30 miRNAs as it was observed with 8 miRNAs earlier. But the difference lies in the repertoire and number of targeted nodes for all these pathways (Table S1 in File S2, Table S2 in File S2, Table S3 in File S2 and Table S4 in File S2).

**Table 6:** Relevant literature search for diseases by 30 significantly deregulated miRNAs in cluster of 13 samples.

Our Observation			Report in previous Publications
Gene	∧p-value	Mean ΔΔCt	Validated Report
hsa-miR-31*	1.00E-10<	−4.96@	MN
hsa-miR-31	5.00E-06	−4.68@	MN,LN, SCC,HN,OLP,EN
hsa-miR-508-3p	6.00E-06	6.66$	Renal Cell Carcinoma
hsa-miR-204	1.30E-05	5.74$	HN metastasis Suppressor
hsa-miR-7	1.60E-05	−3.80@	MN (RECK)
hsa-miR-99a*	1.80E-05	4.03$	HN, Renal Cell (Hitchhike of 99a)
hsa-miR-147b	2.30E-05	−2.55@	Deregulated (similar target with miR-210) (22)
hsa-miR-211	2.40E-05	6.25$	Colorectal (CHD5)
hsa-miR-424*	2.40E-05	−2.71@	Colonic(Abrogate invasive potential)
hsa-miR-369-5p	5.30E-05	3.17$	Reduce proliferation if up regulated (cell cult)
hsa-miR-503	1.31E-04	−2.66@	Endometrial, Hepatocellular, Urin. Blad. Carcinoma
hsa-miR-1247	1.85E-04	3.34$	Cell culture (potential tumor suppressor)
hsa-miR-133a	2.31E-04	7.66$	HN,SCC, LN, EN
hsa-miR-509-5p	2.51E-04	4.13$	Cis-platinum resistance, Down regulation in Adv. Tmr. than Early
hsa-miR-770-5p	2.55E-04	3.64$	-
hsa-miR-337-3p	2.34E-04	4.46$	Lung(STAT3)
hsa-let-7c	3.06E-04	2.5$	Poor responder to chemo
hsa-miR-383	3.08E-04	4.27$	Meduloblastoma
hsa-miR-1293	3.52E-04	−5.03@	*GCN1L1*
hsa-miR-135b*	3.62E-04	−2.78@	Colorectal, LN Hepatocellular, BN (Hitchhike of 135b)
hsa-miR-21	3.96E-04	−2.66@	HN
hsa-miR-139-5p	5.36E-04	2.71$	Stomach
hsa-miR-125b-2*	6.44E-04	3.39$	SCC, LN
hsa-miR-376c	6.98E-04	2.47$	PMID-21224400, Chemo sensitivity
hsa-miR-539	1.12E-03	3.01$	Cell line
hsa-miR-30a-3p	1.14E-03	2.75$	TMEM2, CDK6
hsa-miR-206	1.63E-03	6.24$	NEO-NOTCH3, LN
hsa-miR-411*	1.64E-03	2.8$	Hitchhike
hsa-miR-1	1.70E-03	6.2$	HN
hsa-miR-410	2.40E-03	2.2$	Anti proliferative

@: Up-regulation of expression of miRNAs;

$ : Down regulation of expression of miRNAs.

∧ Benjamini-Hochberg corrected p-value cut off at 5% level: 0.002.

MN- Mouth Neoplasm, HN- Head and Neck Cancer, SCC- Squamous Cell Carcinoma, LN- Laryngeal Neoplasm, EN- Esophageal Neoplasm, OLP-Oral Leukoplakia, BN- Breast Neoplasm, *GCN1L1*- general control of amino-acid synthesis 1-like 1.

## Discussion and Conclusions

Expressions of 7 miRNAs were significantly deregulated in 18 cancer samples and significant up-regulation of *hsa-miR-1293* is being reported for the first time in gingivo buccal cancer (Table 4). As of now, functional role of *hsa-miR-1293* in cancer is very limited. According to miRWalk [21] and StarBase [33], one of the predicted targets of *hsa-miR-1293* is *MAPK14* (p38) which has been shown to be associated with tumor's sensitivity to *cis*-platinum treatment [34]. If this predicted relationship could be validated, then expression of this miRNA may be useful in prediction of patient's sensitivity to *cis*-platinum treatment. Two miRNAs, *hsa-miR-206* and *hsa-miR-1*, are known to play anti-tumorigenic role [35]–[37] and *hsa-miR-*206 may indirectly activate apoptosis, inhibition of cell migration and focus formation [38]. Down regulation of expression of *hsa-miR-1* and *hsa-miR-133a*, which has been observed in this study (Table 5), has already been reported in an earlier study with oral squamous cell carcinoma [39]. In fact, *hsa-miR-133a* targets several oncogenes and is reported to be commonly down regulated in a number of other oral cancer studies [35]–[40]. Expression of both *hsa-miR-31* and hsa-miR-31* was reported in some earlier study on cancer [41]–[42]. Study on OSCC cell line showed that exogenous delivery of *pre-mir-31*, which boosts up quantity of mature *hsa-miR-31* and hsa-miR-31*, enhanced OSCC oncogenicity [41]–[42]. Hence, our observation of up-regulation of *hsa-miR-31* and hsa-miR-31*, corroborates with existing reports on oral and other cancers. Similar to a previous report on head and neck cancer, here also, expression of *hsa-miR-204* was also found to be significantly down regulated [43]. Expression of *hsa-miR-7*, a known OncomiR, is reportedly up-regulated in OSCC [44]. It targets primarily tumor suppressor transcripts from *RECK* [16]. In this study, expression of *hsa-miR-7* was also significantly up-regulated and thus corroborates with previous findings related to oral cancer. Efforts have been made to understand possible biological implication by mining different databases. Pathway analysis revealed that most disrupted biological processes would be cell migration, apoptosis and proliferation (Table S2 in File S2). Most disrupted pathways were predicted to be “proteoglycans in cancer” and *PI3K-AKT* (Table S1 in File S2) which are also supported by our RNA-Seq data on expression deregulation of some proteoglycan genes and *LAMC* and *PAI* which belong to *PI3K-AKT* pathway (unpublished data). These observations also support our predictive pathway analysis regarding involved biological process/pathways that could be targeted by this set of 8 miRNAs.

Cluster analysis of 18 samples had shown that expression of 30 miRNAs was found to be significantly deregulated in the cluster of 13 samples (Figure 3b). Literature search and database mining with these 30 miRNAs showed relevance of these genes with oral and other cancers (Table 6). Although, pathway analysis using 8 miRNAs (identified from expression data of 18 samples) and 30 miRNAs (identified from expression data of cluster of 13 samples) narrowed down to similar signaling pathways but the number and repertoire of nodes targeted by these set of miRNAs are quite different (data not shown). This cluster analysis is actually revealing existence of molecular heterogeneity that may chew up resolution of finding finer molecular marker. Interestingly, observed molecular heterogeneity pattern in no way related to differentiation subtype that exists within the tissues or clinical stages of samples.

Among two pre-cancers, leukoplakia is known to be associated with tobacco habit, whereas lichen planus is an auto immune disease. It is reported that all oral cancers are preceded by precancerous lesions. So, we checked whether, similar to cancer samples, expression deregulation of miRNAs could be observed in these two precancerous lesions. Here, TaqMan data showed that expression of only *hsa-mir-31* was significantly up-regulated (4.55 fold more than adjacent control tissue) in leukoplakia samples (Table 4) but not in lichen planus tissue. Though, expression of another miRNA, hsa-mir-31*, was also up-regulated by 4.75 folds but significantly not different due to greater standard deviation among samples. So, expression of these two miRNAs needs to be checked in more leukoplakia samples. This once again reiterates about molecular proximity of leukoplakia with GBSCC than other pre-cancer, lichen planus. So, expression of hsa-mir-31 and hsa-mir-31* in leukoplakia tissues could be potential risk-markers for progression of precancer to GBSCC.

Small number and mixed stage of tumor samples and expression assay of only 762 miRNAs by TLDA (available at the time of our experiment) limited this study to infer that expression of only 7 miRNAs were significantly deregulated in GBSCC tissues compared to adjacent control tissues. There is high chance that number of deregulated miRNAs will increase if we could assay expression of ∼2578 miRNAs presently known in human tissue as mentioned in miRBase-20 [45]. As a result, expression of more miRNAs could have been checked in cancer and leukoplakia tissues to infer more about molecular markers. More importantly, role of these miRNAs in carcinogenesis is to be validated by functional study.

## Supporting Information

File S1 Genome wide miRNA expression profile for 528 miRNAs (excluding 3 endogenous controls) in GBSCC. Each column from S1 to S24 represents data for 18 samples, each row represents data for ΔΔCt of a specific miRNA. N/A represents no data.
(XLSX)

File S2 Contains supplementary tables.
(DOCX)
