# Supplementary material for: A Quest for miRNA Bio-Marker: A Track Back Approach from Gingivo Buccal Cancer to Two Different Types of Precancers
Source: PLoS One. 2014 Aug 15;9(8):e104839. doi: 10.1371/journal.pone.0104839 (PMC4134240; doi:10.1371/journal.pone.0104839)
Supplement: File S2 — Contains supplementary tables. (DOCX) [file pone.0104839.s002.docx]

**Table S1**: **KEGG pathway mapping with the 702 validated target genes of 8 deregulated miRNAs**

| **KEGG gene list: 8 miRNAs and their 702 targets** |  |  |
| --- | --- | --- |
| hsa05206 MicroRNAs in cancer - Homo sapiens (human) (38*) | | |
| hsa05205 Proteoglycans in cancer - Homo sapiens (human) (37) | | |
| hsa04151 PI3K-Akt signaling pathway - Homo sapiens (human) (35) | | |
| hsa05200 Pathways in cancer - Homo sapiens (human) (35) | | |
| hsa04810 Regulation of actin cytoskeleton - Homo sapiens (human)(28) | | |
| hsa04510 Focal adhesion - Homo sapiens (human) (28) | | |
| hsa04014 Ras signaling pathway - Homo sapiens (human) (23) | | |
| hsa05202 Transcriptional misregulation in cancer - Homo sapiens (human) (21) | | |
| hsa05166 HTLV-I infection - Homo sapiens (human) (19) | | |
| hsa04060 Cytokine-cytokine receptor interaction - Homo sapiens (human) (18) | | |
| hsa04010 MAPK signaling pathway - Homo sapiens (human) (16) | | |
| hsa04062 Chemokine signaling pathway - Homo sapiens (human) (16) | | |
| hsa05169 Epstein-Barr virus infection - Homo sapiens (human) (16) | | |
| hsa05203 Viral carcinogenesis - Homo sapiens (human) (15) | | |
| hsa05215 Prostate cancer - Homo sapiens (human) (15) | | |
| hsa04915 Estrogen signaling pathway - Homo sapiens (human) (14) | | |
| hsa04141 Protein processing in endoplasmic reticulum - Homo sapiens (human) (14) | | |
| hsa04068 FoxO signaling pathway - Homo sapiens (human) (14) | | |
| hsa04921 Oxytocin signaling pathway - Homo sapiens (human) (13) | | |
| hsa04668 TNF signaling pathway - Homo sapiens (human) (13) | | |
| hsa04520 Adherens junction - Homo sapiens (human) (13) | | |
| hsa04012 Erbβ signaling pathway - Homo sapiens (human) (12) | | |
| hsa04210 Apoptosis - Homo sapiens (human) (12) | | |
| hsa05100 Bacterial invasion of epithelial cells - Homo sapiens (human) (12) | | |
|  | | |

*, Number of target genes in each pathway is mentioned in parenthesis

**Table S2**: **DAVID; “GO” term enrichment; Biological process by 702 validated target genes of 8 deregulated miRNAs**

| **Category** | | **Term** | **Count** | | | **%** | **p-Value** | ***Corrected p-value** | |
| --- | --- | --- | --- | --- | --- | --- | --- | --- | --- |
| GOTERM_BP_FAT | positive regulation of macromolecule metabolic process | | | 68 | 0.8 | | 1.30E-07 | | 3.80E-04 |
| GOTERM_BP_FAT | positive regulation of biosynthetic process | | | 57 | 0.7 | | 5.80E-07 | | 8.50E-04 |
| GOTERM_BP_FAT | positive regulation of cellular biosynthetic process | | | 56 | 0.7 | | 8.20E-07 | | 8.10E-04 |
| GOTERM_BP_FAT | negative regulation of apoptosis | | | 36 | 0.4 | | 9.60E-07 | | 7.10E-04 |
| GOTERM_BP_FAT | positive regulation of macromolecule biosynthetic process | | | 54 | 0.7 | | 1.00E-06 | | 5.90E-04 |
| GOTERM_BP_FAT | transmembrane receptor protein tyrosine kinase signaling pathway | | | 27 | 0.3 | | 1.30E-06 | | 6.30E-04 |
| GOTERM_BP_FAT | negative regulation of programmed cell death | | | 36 | 0.4 | | 1.30E-06 | | 5.60E-04 |
| GOTERM_BP_FAT | negative regulation of cell death | | | 36 | 0.4 | | 1.40E-06 | | 5.20E-04 |
| GOTERM_BP_FAT | skeletal system development | | | 33 | 0.4 | | 2.00E-06 | | 6.70E-04 |
| GOTERM_BP_FAT | regulation of apoptosis | | | 61 | 0.8 | | 2.80E-06 | | 8.40E-04 |
| GOTERM_BP_FAT | anti-apoptosis | | | 25 | 0.3 | | 3.10E-06 | | 8.30E-04 |
| GOTERM_BP_FAT | positive regulation of nitrogen compound metabolic process | | | 52 | 0.6 | | 3.20E-06 | | 7.80E-04 |
| GOTERM_BP_FAT | regulation of programmed cell death | | | 61 | 0.8 | | 3.80E-06 | | 8.60E-04 |
| GOTERM_BP_FAT | positive regulation of transcription, DNA-dependent | | | 42 | 0.5 | | 4.30E-06 | | 9.00E-04 |
| GOTERM_BP_FAT | regulation of cell death | | | 61 | 0.8 | | 4.30E-06 | | 8.50E-04 |
| GOTERM_BP_FAT | positive regulation of RNA metabolic process | | | 42 | 0.5 | | 5.30E-06 | | 9.70E-04 |
| GOTERM_BP_FAT | regulation of cellular protein metabolic process | | | 40 | 0.5 | | 2.00E-05 | | 3.50E-03 |
| GOTERM_BP_FAT | positive regulation of gene expression | | | 46 | 0.6 | | 2.10E-05 | | 3.50E-03 |
| GOTERM_BP_FAT | enzyme linked receptor protein signaling pathway | | | 32 | 0.4 | | 2.30E-05 | | 3.60E-03 |
| GOTERM_BP_FAT | positive regulation of nucleobase, nucleoside, nucleotide and nucleic acid metabolic process | | | 48 | 0.6 | | 2.80E-05 | | 4.20E-03 |
| GOTERM_BP_FAT | regulation of phosphorylation | | | 39 | 0.5 | | 3.20E-05 | | 4.40E-03 |
| GOTERM_BP_FAT | positive regulation of cell motion | | | 15 | 0.2 | | 3.80E-05 | | 5.10E-03 |
| GOTERM_BP_FAT | blood vessel morphogenesis | | | 23 | 0.3 | | 4.50E-05 | | 5.80E-03 |
| GOTERM_BP_FAT | positive regulation of transcription | | | 44 | 0.5 | | 4.80E-05 | | 5.80E-03 |
| GOTERM_BP_FAT | negative regulation of macromolecule metabolic process | | | 53 | 0.7 | | 5.40E-05 | | 6.40E-03 |
| GOTERM_BP_FAT | regulation of phosphate metabolic process | | | 39 | 0.5 | | 7.40E-05 | | 8.40E-03 |
| GOTERM_BP_FAT | regulation of phosphorus metabolic process | | | 39 | 0.5 | | 7.40E-05 | | 8.40E-03 |
| GOTERM_BP_FAT | regulation of transcription from RNA polymerase II promoter | | | 52 | 0.6 | | 8.30E-05 | | 9.10E-03 |
| GOTERM_BP_FAT | regulation of cell proliferation | | | 55 | 0.7 | | 9.10E-05 | | 9.50E-03 |

*, Benjamini-Hochberg corrected p-value after multiple testing

**Table S3**: KEGG pathway mapping with the 1207 target genes of 30 significantly deregulated miRNA of 13 sample’s cluster

| **KEGG gene list: 30 miRNAs and their 1207 targets** |  |
| --- | --- |
| hsa01100 Metabolic pathways - Homo sapiens (human) (87*) | |
| hsa05206 MicroRNAs in cancer - Homo sapiens (human) (72) | |
| hsa05200 Pathways in cancer - Homo sapiens (human) (70) | |
| hsa04151 PI3K-Akt signaling pathway - Homo sapiens (human) (63) | |
| hsa05205 Proteoglycans in cancer - Homo sapiens (human) (56) |  |
| hsa04510 Focal adhesion - Homo sapiens (human) (45) |  |
| hsa04810 Regulation of actin cytoskeleton - Homo sapiens (human) (39) |  |
| hsa04010 MAPK signaling pathway - Homo sapiens (human) (39) |  |
| hsa05203 Viral carcinogenesis - Homo sapiens (human) (38) |  |
| hsa04014 Ras signaling pathway - Homo sapiens (human) (36) |  |
| hsa05202 Transcriptional misregulation in cancer - Homo sapiens (human) (36) |  |
| hsa05215 Prostate cancer - Homo sapiens (human) (30) |  |
| hsa05169 Epstein-Barr virus infection - Homo sapiens (human) (28) |  |
| hsa04015 Rap1 signaling pathway - Homo sapiens (human) (27) |  |
| hsa04068 FoxO signaling pathway - Homo sapiens (human) (27) |  |
| hsa04144 Endocytosis - Homo sapiens (human) (26) |  |
| hsa04060 Cytokine-cytokine receptor interaction - Homo sapiens (human) (26) |  |
| hsa04062 Chemokine signaling pathway - Homo sapiens (human) (26) |  |
| hsa04390 Hippo signaling pathway - Homo sapiens (human) (26) |  |
| hsa04110 Cell cycle - Homo sapiens (human) (25) |  |
| hsa05168 Herpes simplex infection - Homo sapiens (human) (25) |  |
| hsa04066 HIF-1 signaling pathway - Homo sapiens (human) (25) |  |
| hsa05222 Small cell lung cancer - Homo sapiens (human) (24) |  |
| hsa04668 TNF signaling pathway - Homo sapiens (human) (23) |  |
| hsa05214 Glioma - Homo sapiens (human) (23) |  |
| hsa04910 Insulin signaling pathway - Homo sapiens (human) (22) |  |
| hsa04141 Protein processing in endoplasmic reticulum - Homo sapiens (human) (22) |  |
| hsa04630 Jak-STAT signaling pathway - Homo sapiens (human) (22) |  |
| hsa05220 Chronic myeloid leukemia - Homo sapiens (human) (22) |  |
| hsa05212 Pancreatic cancer - Homo sapiens (human) (22) |  |
| hsa04115 p53 signaling pathway - Homo sapiens (human) (22) |  |

*, Number of target genes in each pathway is mentioned in parenthesis

**Table S4:** **DAVID; “GO” term enrichment; Biological process on 1207 validated target genes of 30 deregulated miRNAs. Top 30 biological processes are only shown**

| **Category** | | **Term** | **Count** | | | **%** | | **P-Value** | | ***Corrected p- value** | |
| --- | --- | --- | --- | --- | --- | --- | --- | --- | --- | --- | --- |
| GOTERM_BP_FAT | positive regulation of macromolecule metabolic process | | | 124 | 0.4 | | 6.90E-15 | | 2.50E-11 | |  |
| GOTERM_BP_FAT | positive regulation of cellular biosynthetic process | | | 101 | 0.3 | | 1.10E-12 | | 2.00E-09 | |  |
| GOTERM_BP_FAT | positive regulation of biosynthetic process | | | 102 | 0.3 | | 1.10E-12 | | 1.40E-09 | |  |
| GOTERM_BP_FAT | positive regulation of nitrogen compound metabolic process | | | 96 | 0.3 | | 2.30E-12 | | 2.10E-09 | |  |
| GOTERM_BP_FAT | positive regulation of macromolecule biosynthetic process | | | 97 | 0.3 | | 2.40E-12 | | 1.70E-09 | |  |
| GOTERM_BP_FAT | positive regulation of transcription, DNA-dependent | | | 78 | 0.3 | | 3.70E-12 | | 2.20E-09 | |  |
| GOTERM_BP_FAT | positive regulation of RNA metabolic process | | | 78 | 0.3 | | 5.60E-12 | | 2.90E-09 | |  |
| GOTERM_BP_FAT | regulation of programmed cell death | | | 110 | 0.4 | | 2.20E-11 | | 1.00E-08 | |  |
| GOTERM_BP_FAT | positive regulation of gene expression | | | 87 | 0.3 | | 2.30E-11 | | 9.30E-09 | |  |
| GOTERM_BP_FAT | regulation of apoptosis | | | 109 | 0.4 | | 2.60E-11 | | 9.60E-09 | |  |
| GOTERM_BP_FAT | regulation of cell death | | | 110 | 0.4 | | 2.80E-11 | | 9.10E-09 | |  |
| GOTERM_BP_FAT | positive regulation of transcription | | | 84 | 0.3 | | 7.10E-11 | | 2.20E-08 | |  |
| GOTERM_BP_FAT | positive regulation of nucleobase, nucleoside, nucleotide and nucleic acid metabolic process | | | 90 | 0.3 | | 7.50E-11 | | 2.10E-08 | |  |
| GOTERM_BP_FAT | negative regulation of apoptosis | | | 61 | 0.2 | | 1.30E-10 | | 3.50E-08 | |  |
| GOTERM_BP_FAT | negative regulation of programmed cell death | | | 61 | 0.2 | | 2.40E-10 | | 5.80E-08 | |  |
| GOTERM_BP_FAT | negative regulation of cell death | | | 61 | 0.2 | | 2.70E-10 | | 6.10E-08 | |  |
| GOTERM_BP_FAT | negative regulation of macromolecule metabolic process | | | 99 | 0.3 | | 3.40E-10 | | 7.20E-08 | |  |
| GOTERM_BP_FAT | regulation of cell proliferation | | | 102 | 0.3 | | 1.60E-09 | | 3.20E-07 | |  |
| GOTERM_BP_FAT | phosphorus metabolic process | | | 119 | 0.4 | | 1.90E-09 | | 3.70E-07 | |  |
| GOTERM_BP_FAT | phosphate metabolic process | | | 119 | 0.4 | | 1.90E-09 | | 3.70E-07 | |  |
| GOTERM_BP_FAT | regulation of transcription from RNA polymerase II promoter | | | 96 | 0.3 | | 2.00E-09 | | 3.70E-07 | |  |
| GOTERM_BP_FAT | vasculature development | | | 46 | 0.1 | | 4.80E-09 | | 8.30E-07 | |  |
| GOTERM_BP_FAT | regulation of phosphorylation | | | 69 | 0.2 | | 5.80E-09 | | 9.50E-07 | |  |
| GOTERM_BP_FAT | phosphorylation | | | 101 | 0.3 | | 7.90E-09 | | 1.30E-06 | |  |
| GOTERM_BP_FAT | regulation of kinase activity | | | 57 | 0.2 | | 1.00E-08 | | 1.60E-06 | |  |
| GOTERM_BP_FAT | anti-apoptosis | | | 40 | 0.1 | | 1.10E-08 | | 1.60E-06 | |  |
| GOTERM_BP_FAT | regulation of cellular protein metabolic process | | | 69 | 0.2 | | 1.20E-08 | | 1.60E-06 | |  |
| GOTERM_BP_FAT | regulation of phosphate metabolic process | | | 70 | 0.2 | | 1.30E-08 | | 1.70E-06 | |  |
| GOTERM_BP_FAT | regulation of phosphorus metabolic process | | | 70 | 0.2 | | 1.30E-08 | | 1.70E-06 | |  |
| GOTERM_BP_FAT | positive regulation of transcription from RNA polymerase II promoter | | | 58 | 0.2 | | 1.60E-08 | | 2.10E-06 | |  |

*, Benjamini-Hochberg corrected p-value after multiple testing
